# The association between sedentary behaviour and indicators of stress: a systematic review

**DOI:** 10.1186/s12889-019-7717-x

**Published:** 2019-10-23

**Authors:** Megan Teychenne, Lena D. Stephens, Sarah A. Costigan, Dana Lee Olstad, Brendon Stubbs, Anne I. Turner

**Affiliations:** 1 Institute for Physical Activity and Nutrition (IPAN), School of Exercise and Nutrition Sciences, Deakin University, Geelong, Australia; 2 School of Exercise and Nutrition Sciences, Deakin University, Geelong, Australia; 3 Department of Community Health Sciences, Cumming School of Medicine, University of Calgary, Calgary, Canada; 4 Institute of Psychiatry, Psychology and Neuroscience, King’s College London, London, UK

**Keywords:** Sedentary behaviour, Sitting time, Television viewing, Stress, Mental health, Adults

## Abstract

**Background:**

Emerging evidence shows sedentary behaviour may be associated with mental health outcomes. Yet, the strength of the evidence linking sedentary behaviour and stress is still unclear. This study aimed to synthesise evidence regarding associations between time spent in sedentary behaviour and stress in adults.

**Methods:**

A systematic search was conducted (January 1990 – September 2019). Following PRISMA guidelines, an evaluation of methodological quality, and best-evidence synthesis of associations between time in sedentary behaviour (including sitting time, TV viewing, computer use) and stress were presented. Twenty-six studies reporting on data from *n* = 72,795 people (age 18-98y, 62.7% women) were included.

**Results:**

Across the studies (*n* = 2 strong-, *n* = 10 moderate- and *n* = 14 weak-quality), there was insufficient evidence that overall time spent in sedentary behaviour and sitting time were associated with stress, particularly when using self-report measures of sedentary behaviour or stress. There was strong evidence of no association between TV viewing, or computer use and stress. Amongst studies using objective measures of sedentary behaviour and/or stress there was also strong evidence of no association.

**Conclusion:**

Although previous research suggested sedentary behaviour may be linked to mental health outcomes such as depression and anxiety, the evidence for an association between various types of sedentary behaviour and stress is limited in quality, and associations are either inconsistent or null. High-quality longitudinal/interventional research is required to confirm findings and determine the direction of associations between different contexts (i.e. purpose) and domains (i.e. leisure, occupational, transport) of sedentary behaviour and stress.

## Background

Psychological stress is a negative emotional state associated with nervousness, tension and/or strain [1, 2], which can be characterised by feelings of worry, fatigue and inability to cope [3]. Stress has been described as the ‘modern day hidden epidemic’ [2], due to its high and rising prevalence and impact on health worldwide. Approximately 16% of adults in Sweden [4] and Australia [5], 22% in Europe [6] and 24% in the United States (US) [7] report moderate to severe perceived stress. Chronic and/or high levels of stress are associated with increased risk of adverse health conditions, including cardiovascular disease and events [8], depression [9] and long-term disability [10]. Further, psychological stress is linked to reduced workplace productivity and increased absenteeism [11] and is estimated to cost the US USD$42 billion [2], the EU-15 €26.47 billion [12], and Australia AUD$25 billion [13] per year. Given this significant impact, understanding lifestyle factors that may influence (or be influenced by) stress is essential.

There is growing evidence showing that positive lifestyle behaviours such as increased physical activity/exercise [14, 15], a healthy diet [16] and smoking cessation [17] are linked to reduced risk of psychological stress, as well as other mental health outcomes such as depression and anxiety symptoms [17–19]. However, less is known regarding the relationship between sedentary behaviour (i.e. sitting or reclining behaviours requiring minimal energy expenditure [20]) and stress. Given that there is an increasing evidence base to suggest that sedentary behaviour (such as computer use, TV viewing, and overall sitting) is linked to poor mental health, specifically depression [21] and anxiety [22], it is plausible that sedentary behaviour may also be linked to stress.

Theoretically, sedentary behaviour could be associated with stress via a number of potential pathways. For example, screen-based sedentary behaviours such as TV viewing, computer or electronic device use (i.e. smartphones, tablets) can induce feelings of addiction [23], ‘brain burnout’ [5] and/or sleeping problems [24], potentially leading to heightened levels of psychological stress. Further, engaging in such sedentary behaviours may displace time spent in other important activities such as undertaking household or work-related responsibilities, or physical activity, which may then increase feelings of stress [25]. Alternatively, since TV viewing is a popular strategy used by many adults in developed countries to manage stress (e.g. reported by 85% of respondents from the Stress and Wellbeing in Australia Survey, *n* = 1521; and 39% in the Stress in America Survey, *n* = 3361) [5], it is possible that some sedentary behaviours may reduce stress. Yet, there is a lack of clarity regarding associations between different types of sedentary behaviour and stress, and no previous review has summarised the evidence to date. Thus, the aim of this review was to investigate associations between time spent in sedentary behaviour and stress in adults.

## Methods

The protocol was registered with PROSPERO: https://www.crd.york.ac.uk/PROSPERO/ (registration number: CRD42018091235).

### Search strategy

A systematic electronic search was undertaken for articles published from January 1990 to September 2019. Findings are reported according to PRISMA reporting guidelines [26]. Articles prior to 1990 were not included as increases in population sedentary behaviour levels did not begin to be reported until after 1990 with the advent of widespread online technology use [27]. Databases included Medline/Medline Complete, CINAHL Complete, PsychINFO, SPORTDiscus and EMBASE. Full search strings are provided in Additional file 1: Table S1, however, principal search terms encompassed: 1) ‘Sedentary behaviour’ (sedentary behaviour, screen time, screen-based, television, computer, electronic device, video game, smart phone, sitting, passive transport, and tablet); and 2) ‘Stress’ (stress, cortisol, adrenocortical/glucocorticoid hormones). Search strings were further limited to peer-reviewed articles written in English. The literature search, and each of the following stages was led by LDS. Duplicates were identified by LDS in two stages. The majority of duplicates were first identified using EndNote’s automated ‘find duplicates’ function. Remaining duplicates were then identified in EndNote by sorting article titles alphabetically before manual checking, then sorting author names alphabetically before again checking manually. After removal of duplicates, article titles were initially screened for inclusion by LDS and MT. Abstracts were then assessed, followed by retrieval of full texts which were read to determine suitability. Reference lists from retrieved full-text articles and authors’ own bibliographic libraries were also searched, yielding no additional articles. MT and LDS reviewed all final articles to determine their inclusion in the review.

### Study selection criteria

For the purpose of this review, stress was operationalised as both self-reported (e.g. perceived psychological stress) and objective (e.g. changes in stress hormones such as cortisol) measures of stress. Articles were eligible for inclusion if they: 1) were published in a peer-reviewed journal in English between January 1990 to September 2019; 2) examined apparently healthy adults aged ≥18 years (i.e. those not specifically recruited among populations with underlying chronic physical conditions (e.g. diabetes) or mental disorders (e.g. depression)); 3) examined self-reported or objective measures of screen-based sedentary behaviour or other forms of sitting time; 4) assessed self-reported or objective measures of stress; and 5) employed a cross-sectional, longitudinal, direct observation (e.g. where time in sedentary behaviour was collected for one year or one month and averaged), or controlled experimental study design. Qualitative studies and review articles were excluded. Intervention studies that primarily investigated the direct relationship between sedentary behaviour and a measure of stress were eligible to be included; however, studies that reported the effect of an intervention on stress, or sedentary behaviour, independent of one another, were not eligible. Due to potential confounding, articles relating to yoga or meditation for stress relief, or that measured stress in relation to engagement in violent screen-based activities (e.g. viewing violent TV shows, playing violent computer games) were excluded from the review, as were conference abstracts, dissertations, theses, and articles published in non-peer-reviewed journals.

### Data extraction

Data extraction was performed by LDS using a data extraction form that was pilot-tested initially by MT, SAC and LDS on three included articles. Key study characteristics were extracted from identified studies included country in which the study took place, study population characteristics (sample size, age, and sex of participants), study design, sedentary behaviour type (e.g. screen-time, sitting time) and measures (e.g. self-reported or objective [i.e. direct observation or device assessed]) of sedentary behaviour, measures of stress (e.g. self-reported or objective measures) and study results in terms of the association between sedentary behaviour and stress.

### Methodological quality

Methodological quality of each study was evaluated using the Effective Public Health Practice Project Quality Assessment Tool for Quantitative Studies – recommended by the Cochrane Public Health Review Group [28]. The tool assesses research studies on selection bias (e.g. representativeness, response rate), study design (e.g. longitudinal, randomized controlled trial), confounders (e.g. controlling for confounders such as sociodemographic characteristics), blinding (e.g. researcher/participant awareness of group allocation), data collection methods (e.g. validity and reliability of measures), withdrawals and dropouts (e.g. reasons, proportion of sample with complete data), intervention integrity (e.g. percent receiving intervention) and analyses (e.g. appropriate statistical analyses for study design). For all studies, each component was given an overall quality score of weak, moderate, or strong, following the established protocol [28]. An overall study rating was assigned to each study as follows: weak (if they received≥2 weak ratings); moderate (if they received one weak rating); or strong (if they received no weak ratings). The methodological quality of studies was independently assessed by two reviewers (LDS and SAC). Any discrepancies were resolved via discussion.

### Best-evidence synthesis

To enable conclusions on associations between sedentary behaviour and stress on the basis of the methodological quality of studies, a best-evidence synthesis [29] was conducted by two authors (MT and LDS). Due to the heterogeneity in exposure and outcomes, a best-evidence synthesis was selected over a meta-analysis given it would not be meaningful to calculate the average effect as per a meta-analysis [29, 30]. This method has been used previously in systematic reviews in the area of sedentary behaviour and health outcomes [22, 31, 32]. Adapted from guidelines outlined in previous reviews that applied best-evidence synthesis [22, 31], the evidence was graded as strong, moderate, or insufficient. Consistency was defined on two levels: 1) *within a study* (i.e. ≥75% of results in same direction within a study), to account for multiple modelling; and 2) *between studies* (i.e. ≥75% of results in same direction across studies examined). **Strong evidence** was defined as consistent results in ≥2 strong/moderate quality studies. **Moderate evidence** was defined as consistent results in one strong/moderate quality study and at least one weak-quality study; or consistent results in ≥2 weak-quality studies. **Insufficient evidence** was defined as having only one available study or *inconsistent results* in ≥2 studies. When ≥2 studies were of strong/moderate methodological quality, those with weak-quality were disregarded in the evidence synthesis [31]. In this manner, evidence was weighted in terms of study design/methodological quality.

To determine whether associations between time in sedentary behaviour and stress could be explained by the nature of the sedentary behaviour and stress measures, studies were grouped and results analysed firstly on the basis of utilising objective versus self-report measures of stress, and secondly, on the use of objective (i.e. device assessed or direct observation) and self-report measures of sedentary behaviour.

## Results

**Fig. 1:**
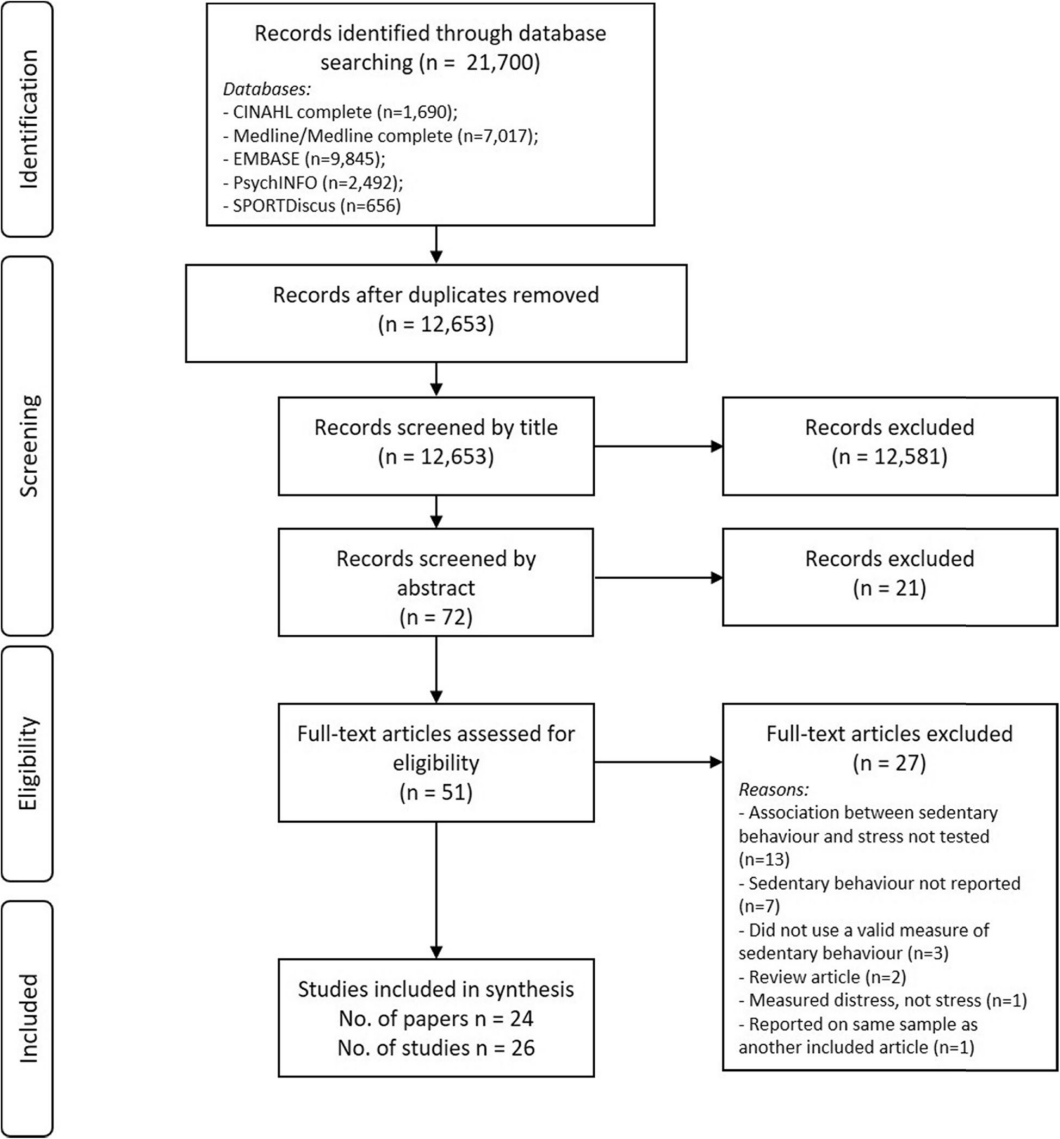
PRISMA Flow diagram of study selection

Literature searching yielded 12,653 articles after duplicates were removed (Fig. 1), which were screened by title. After further screening of abstracts (*n* = 72), and full papers (*n* = 51), a total of 26 studies (reported in 24 papers – note: Anderson et al. (1996) included three studies presented within the one paper [33]) were included in the review. Characteristics of included studies are summarised in Table 1. Sixteen studies employed a cross-sectional design [33–36, 41, 43, 44, 46, 47, 49–51, 53, 54, 56, 57] (*n* = 72 to 34,129 participants). Four studies were longitudinal [33, 38, 45, 55] (*n* = 271 to 11,676), two were controlled clinical trials [39, 48] (*n* = 43–231), two were pilot interventions [40, 52] (*n* = 12 to 20) and two were direct observation studies (i.e. where time in sedentary behaviour was collected for one year or one month and averaged) [33, 37] (*n* = 79 to 140). The majority of these studies were conducted in Australia (*n* = 8) and the US (*n* = 8). Twenty one of the 26 study samples were comprised of men and women (age 18-98y) [33–41, 43, 44, 47–52, 56, 57], while the remaining five studies were conducted among women only (age 18-65y) [45, 46, 53–55].

**Table 1:** Characteristics of studies investigating associations between sedentary behaviour and stress (alphabetically ordered)

Authors (date) and country	Study design & sample	Sedentary behaviour indicator	Stress indicator	Association (and direction)	Consistency in findings i.e. ≥75% of results in same direction	Methodological quality score
An, Jang and Kim (2015) [34] Republic of Korea	Cross-sectional *n* = 4674 Age: ≥20y 58.5% women	Self-report: Total daily sitting (including sitting at work, home, studying and during leisure time); Korean version of IPAQ-SV.	Self-report: Stress symptoms: “Have you felt sad/desperate to the extent it disturbed daily life for more than two weeks during the past year, so much that it disturbed your daily life?”	Total sitting = +	Sitting = +	Moderate
Anderson, et al. (1996) [33] US	*Study A* Cross sectional *n* = 491 Age: 18-88y 54.8% women	Self-report: Total weekly TV viewing.	Self-report: 51-item Life Events Inventory.	TV = 0	TV = 0	Weak
Anderson, et al. (1996) [33] US	*Study B* Longitudinal *n* = 651 Age: me*n* = 34.8y, women = 32.8y 50.5% women	Self-report: Weekly TV viewing (calculated from two 10-day TV viewing diaries, recorded one month apart).	Self-report: 51-item Life Events Inventory.	TV (women) = + TV (men) = 0	TV = +/0	Weak
Anderson, et al. (1996) [33] US	*Study C* Direct observation and longitudinal (survey) *n* = 140 Age: mid 20s to late 30s 50.7% women	Objective: Time-lapse video of TV and TV viewing areas recorded for 10 continuous days to ascertain when participant was looking at the TV.	Self-report: 51-item Life Events Inventory.	TV (men) = + TV (women) = 0	TV = +/0	Weak
Ashdown-Franks, et al. (2018) [35] China, Ghana, India, Mexico, Russia and South Africa	Cross-sectional n = 34,129 Age: ≥50y (mean ± SD = 62.4y ± 16.0) 55.0% women	Self-report: Total mins/day spent sitting/ reclining.	Self-report: Two items of the Cohen Perceived Stress Scale.	Total sitting (adjusted) = + Total sitting (50-64y) = + Total sitting (≥65y) = +	Sitting = +	Moderate
Depp, et al. (2010) [36] US	Cross-sectional *n* = 3982 Age: 15-98y (mean ± SD = 51.4 ± 18.0) 61.0% women	Self-report: 15 min ‘episodes’ engaged in TV viewing, as defined in the American Time-Use Survey lexicon.	Self-report: Affective experience: ‘Feeling stressed’ (from the Princeton Affect and Time Survey).	TV = −	TV = −	Weak
Diaz, et al. (2018) [37] US	Direct observation *n* = 79 Age: mean ± SD = 31.9y ± 9.5 14.1% women	Objective: Accelerometry. Total sitting time (min/day), and mean sitting bout duration (min/bout).	Self-report: Participants recorded momentary stress (prompted randomly three times/day), and end-of-day (prompted in evening, once/day). Prompts based on Daily Stress Inventory (including work, argument, traffic, deadlines, bills, running late, or other).	Total sitting time: Work = 0 Argument = + Traffic jam = 0 Deadlines = 0 Bills = 0 Running late = − Other stress = 0	Sitting = 0	Weak
Ellingson, et al. (2018) [38] US	Longitudinal *n* = 271 Age: mean ± SD = 27.8y ± 3.7 49.0% women	Objective: Accelerometry. Total sedentary time (hrs/day); and low (< 10.5 h), medium (10.5–12 h), and high (> 12 h) sub-groups. Sedentary bout duration (< 30 min, ≥30 min).	Self-report: Ten-item Cohen Perceived Stress Scale.	Baseline: Total sitting = 0 Stratified by sitting sub-group = 0 Sitting bout duration < 30 min = 0 Sitting bout duration ≥30 min = 0 Total sitting Δ over time = + (as sedentary behaviour increased over time, stress increased) Total sitting Δ over time (stratified by baseline sedentary behaviour sub-group): Low = + Medium = + High = + (as sedentary behaviour increased over time, stress increased) Sitting bout duration Δ over time: < 30 min = 0 ≥30 min = +	Sitting = +/0	Weak
Endrighi, Steptoe and Hamer (2016) [39] UK	Intervention *n* = 43 Age mean ± SD: 23.86y ± 4.71 (men), 25.73y ± 0.13 (women) 44.2% women	Objective: Accelerometry. Change in total sedentary time (min/day) between treatment (sedentary) and control (usual behaviour) conditions.	Objective: Systolic and diastolic blood pressure, heart rate, salivary cortisol	Total sitting (systolic and diastolic blood pressure) = 0 Total sitting (heart rate) = 0 Total sitting (salivary cortisol) = 0	Sitting = 0	Moderate
Gilson, et al. (2017) [40] Australia	Pilot intervention *n* = 20 Age: mean ± SD = 37.9 ± 11.6 55.0% women	Objective: Observed three 1.5 h work periods per treatment group: 1) usual chair and desk use, 2) sit–stand desk, and 3) treadmill desk.	Objective: Salivary cortisol.	Occupational sitting (usual chair and desk use) = +	Sitting = +	Weak
Gubelmann, et al. (2018) [41] Switzerland	Cross-sectional *n* = 1948 Age: 45-86y 55.0% women	Objective: Accelerometry. Mean time (%) spent sitting (all days). Mean sitting stratified into tertiles, classified as ‘high sedentary behaviour’ if in the highest tertile, and as ‘low sedentary behaviour’ if in the remaining tertiles.	Objective: Salivary cortisol measured at T1 (waking), T2 (30 min after T1), T3 (11:00 am) and T4 (8:00 pm). Mean cortisol and diurnal cortisol slope (Steeper decline tends to be associated with more favourable health outcomes [42]; T4-T1 cortisol divided by number of hours separating T1-T4).	Low vs high mean sitting: Mean cortisol: Adjusted = 0 Awakening cortisol: Adjusted = 0 Diurnal cortisol slope: Adjusted = +	Sitting = 0/+	Moderate
Jackson, et al. (2019) [43] UK	Cross-sectional *n* = 3555 Age: ≥50y (mean ± SD = 68.34 ± 7.86) 66.6% women	Self-report: Mean daily hours TV viewing (combined weekdays and weekend days); < 2 h/day; 2 to< 4 h/day; 4 to< 6 h/day; ≥6 h/day.	Objective: Hair cortisol.	TV (adjusted) = 0	TV = 0	Moderate
Lee and Kim (2018) [44] Republic of Korea	Cross-sectional *n* = 244 Age: University students (age not reported) 80.0% women	Self-report: Mean hours/day spent engaged in activities that do not increase energy expenditure above resting, i.e. ~ 1.0–1.5 METs (total, week and weekend days).	Self-report: Ten-item Cohen Perceived Stress Scale.	Total sitting (adjusted) = + Week day sitting (adjusted) = + Weekend day sitting (adjusted) = 0	Sitting = +/0	Weak
Mouchacca, Abbott and Ball (2013) [45] Australia	Longitudinal *n* = 1382 Age: 18-46y (mean ± SD = 35.7 ± 7.7) 100.0% women	Self-report (T1 and T2): Total weekly hours spent sitting (IPAQ-L) and total weekly TV viewing.	Self-report (T1): Four-item Perceived Stress Scale.	TV (baseline) = 0 TV (at follow-up) = + Total weekly sitting (baseline) = 0 Total weekly sitting (follow-up) = 0	TV = 0/+ Sitting = 0 ^*a*^ Overall SB = 0	Strong
Pavić and Rijavec (2013) [46] Croatia	Cross-sectional *n* = 216 Age: 18-45y (mean ± SD = 26.51 ± 7.63) 100.0% women	Self-report: Total weekly TV viewing.	Self-report: 10-item version of the Cohen Perceived Stress Scale and sub-scales ‘Negative emotions’ and ‘Lack of control’.	TV (overall stress) = + TV (‘Negative emotions’) = + TV (‘Lack of control’) = +	TV = +	Weak
Pelletier, Lytle, and Laska (2016) [47] US	Cross-sectional *n* = 441 Age: <21y (50.6%), ≥21 (49.4%) 67.6% women	Self-report: Total daily sitting and reclining (WHO Global Physical Activity Questionnaire).	Self-report: Four-item Cohen Perceived Stress Scale.	Total daily sitting/reclining = 0	Sitting = 0	Weak
Peterman, et al. (2019) [48] Australia	Intervention *n* = 231 Age: 18-65y (mean ± SD = 45.6 ± 9.4) 68.0% women	Objective: Accelerometry. Change in mean min spent sitting/8-h workday between treatment (reduced workplace sitting) and control (usual working conditions).	Self-report: Single stress item from the Health and Work Questionnaire (HWQ)	Occupational sitting = 0	Sitting = 0	Moderate
Rebar, et al. (2016) [49] Australia	Cross-sectional *n* = 1104 Age: mean = 58y 55.0% women	Self-report: Daily sitting in the following contexts: leisure, occupation, computer use, TV, and transport; and overall sitting time (10-item Workforce Sitting Questionnaire).	Self-report: Depression, Anxiety, and Stress Scale (DASS-21).	Transport sitting = + Leisure = 0 Occupational sitting = 0 Overall sitting time = 0 Computer = 0 TV = 0	Sitting = 0 Computer = 0 TV = 0 ^*a*^ *Overall SB = 0*	Moderate
Ryde, et al. (2019) [50] UK	Cross-sectional *n* = 77 Age: mean ± SD = 40.8 ± 9.7 78.0% women	Objective: Accelerometry (mean min/day)	Objective: Hair cortisol. Self-report: Ten-item Cohen Perceived Stress Scale.	Occupational sitting (objective stress, adjusted) = 0 Occupational sitting (self-report stress, adjusted) = 0	Sitting = 0	Moderate
Sonnentag and Jelden (2009) [51] Germany	Cross-sectional *n* = 78 Age: mean ± SD = 43.8y ± 7.7 14.1% women	Self-report: Overall time daily spent engaged in ‘low effort’ activities (e.g. watching TV, reading newspaper, doing nothing).	Self-report: Job stressors: ‘time pressure’, ‘role ambiguity’ and ‘situational constraints’ (from shortened job stressor scales).	Total sitting (‘situational constraints’) = + Total sitting (‘time pressure’) = 0 Total sitting (‘role ambiguity’) = 0	Sitting = +/0	Weak
Sperlich, et al. (2018) [52] Germany	Pilot intervention *n* = 12 Age: mean ± SD = 22.0y ± 2.0 58.0% women	Objective: Researchers observed participants completing a control or treatment routine. Treatment routine included: T0: Resting lying down for 30 min T1: Consumed breakfast T2: Sitting for one hour T3: Six min HIIT session T4-T7: Sitting for two hours (T4 = 30-, T5 = 60-, T6 = 90-, and T7 = 120-min after HIIT session). Control routine included: T0 and T1 described above, followed by 186 min of sitting.	Objective: Salivary cortisol. Samples collected at T0, T2, T3, T4, T5, T6 and T7.	Compared to baseline (T0) measurement (results presented for control group only): T2: Sitting = − T3: Sitting = − T4: Sitting = − T5: Sitting = − T6: Sitting = − T7: Sitting = −	Sitting = −	Weak
Teychenne, Ball and Salmon (2012) [53] Australia	Cross-sectional *n* = 1554 Age: 18-65y (mean ± SD = 42.0y ± 12.78) 100.0% women	Self-report: Total weekly TV viewing.	Self-report: Four-item Cohen Perceived Stress Scale.	TV = 0	TV = 0	Moderate
Teychenne, et al. (2018) [54] Australia	Cross-sectional *n* = 72 Age:18-46y (mean ± SD = 43.5y ± 7.1) 100.0% women	Self-report: Weekly hours engaged in TV viewing, computer use and overall sitting time.	Objective: Hair cortisol.	TV = 0 Computer = 0 Sitting = 0	TV = 0 Computer = 0 Sitting = 0 ^*a*^ Overall SB = 0	Moderate
Uijtdewilligen, et al. (2014) [55] Australia	Longitudinal n = 11,676 Age (mean ± SD): 2000–24.6y ± 1.5; 2003–27.6y ± 1.5; 2006–30.6y ± 1.5; 2009–33.7y ± 1.5 100.0% women	Self-report: Total daily hours sitting on weekdays, and on weekend days.	Self-report: Perceived Stress Questionnaire for Young Women.	Weekday sitting (multivariate) = + Weekend day sitting (multivariate) = +	Sitting = +	Strong
Vasquez, et al. (2016) [56] US	Cross-sectional *n* = 4244 Age: 18-74y 62.0% women	Objective: Accelerometry. Mean min/day. Self-report: Global Physical Activity Questionnaire	Self-report: Chronic stress (8-item Chronic Burden scale) and traumatic stress (10-item Traumatic Stress Schedule).	Objective sitting: *Chronic stress:* Model 3 = + *Traumatic stress:* Model 3 = + Self-report sitting/reclining: Chronic stress (age and field-centre adjusted) = + Traumatic stress (age and field-centre adjusted) = +	Sitting = +	Weak
Wang, et al. (2018) [57] Australia	Cross-sectional *n* = 1481 Age: 31-41y (mean ± SD: men, 36.8 ± 2.5; women, 36.5 ± 2.6) 58.0% women	Self-report: Mean week day and weekend day sitting time (min/day; IPAQ-L).	Self-report: Effort Reward Imbalance 17-item scale.	Self-report sitting *Week day sitting:* Model 3 (men) = 0 Model 3 (women) = 0 *Weekend day sitting:* Model 3 (men) = + Model 3 (women) = 0	Sitting = 0	Weak

*TV* television; + = increasing sedentary behaviour is associated with increased stress, − = increasing sedentary behaviour is associated with decreased stress, 0 = no association

^a^Overall sedentary behaviour (SB) = composite direction/score for sedentary behaviour, whereby 75% of within-study results reflect an overall direction

Objective measures of stress were used in n = 7 studies, and included salivary [39–41, 52] or hair [43, 50, 54] cortisol, blood pressure (systolic and diastolic) [39] and heart rate [39]. Stress was self-reported using measures including the Life Events Inventory (LEI, *n* = 3) [33], the Cohen Perceived Stress Scale (PSS, *n* = 8) [35, 38, 44–47, 50, 53], the Depression, Anxiety and Stress Scale (DASS-21, *n* = 1) [49], the Perceived Stress Questionnaire for Young Women (PSQYW, *n* = 1) [55], the affective experience component of the Princeton Affect and Time Survey (n = 1) [36], the Daily Stress Inventory (DSI, *n* = 1) [37], the Chronic Burden Scale (CBS, *n* = 1) [56], the Traumatic Stress Schedule (TSS, *n* = 1) [56], job stressors (*n* = 1) [51], the Health and work Questionnaire (*n* = 1) [48] and the Effort Reward Imbalance Scale (ERIS, *n* = 1) [57]. One study measured stress using self-report survey items designed specifically for that study [34].

Ten studies measured sedentary behaviour objectively (*n* = 7 utilised accelerometers [37–39, 41, 48, 50, 56]; *n* = 2 directly observed participants’ sitting time in a laboratory setting [40, 52]; *n* = 1 objectively measured TV viewing time via time-lapse video recordings [33]). Self-report measures of sedentary behaviour were used in 16 studies. Types of sedentary behaviour that were self-reported included: total daily and/or weekly sitting [34, 35, 44, 45, 47, 49, 51, 54–57]; TV viewing time [33, 36, 43, 45, 46, 49, 53, 54]; occupational sitting time [49]; computer use [49, 54]; transport-related sitting time [49]. Reliability and validity of all the above measures of sedentary behaviour and stress are reported in Additional file 2: Table S2.

### Methodological quality

Table 1 presents methodological quality scores. Overall, two studies were rated as strong [45, 55], 10 studies [34, 35, 39, 41, 43, 48–50, 53, 54] were rated as moderate and 14 studies [33, 36–38, 40, 44, 46, 47, 51, 52, 56, 57] received a weak methodological quality rating. Studies received weak or moderate ratings primarily due to study design limitations (e.g. 16 of 26 studies employed a cross-sectional study design [33–36, 41, 43, 44, 46, 47, 49–51, 53, 54, 56, 57]), participants were not likely to be representative of target population (8 of 26 studies) [37, 39, 40, 44, 46, 47, 51, 52], or the studies did not employ/report reliable and/or valid measures for sedentary behaviour (11 of 26 studies) [33–36, 40, 44, 47, 51, 55] (see Additional file 2: Table S2).

Results are presented as a whole sample (i.e. combined findings) first, then for specific sedentary behaviours (i.e. overall sitting time, TV viewing, computer use), and finally for objectively versus subjectively assessed sedentary behaviour and stress.

### Combined findings

Unadjusted results are reported here first, i.e. not adjusting for publication bias, nor for multiple modelling within studies. Overall, across the 26 studies, there were 78 models that assessed the association between sedentary behaviour (any indicator) and stress. This resulted in 29 positive associations (37%; i.e. higher sedentary behaviour associated with higher levels of stress); eight inverse associations (10%; i.e. higher sedentary behaviour associated with lower levels of stress) and 41 null results (53%; i.e. no association between sedentary behaviour and stress).

**Fig. 2:**
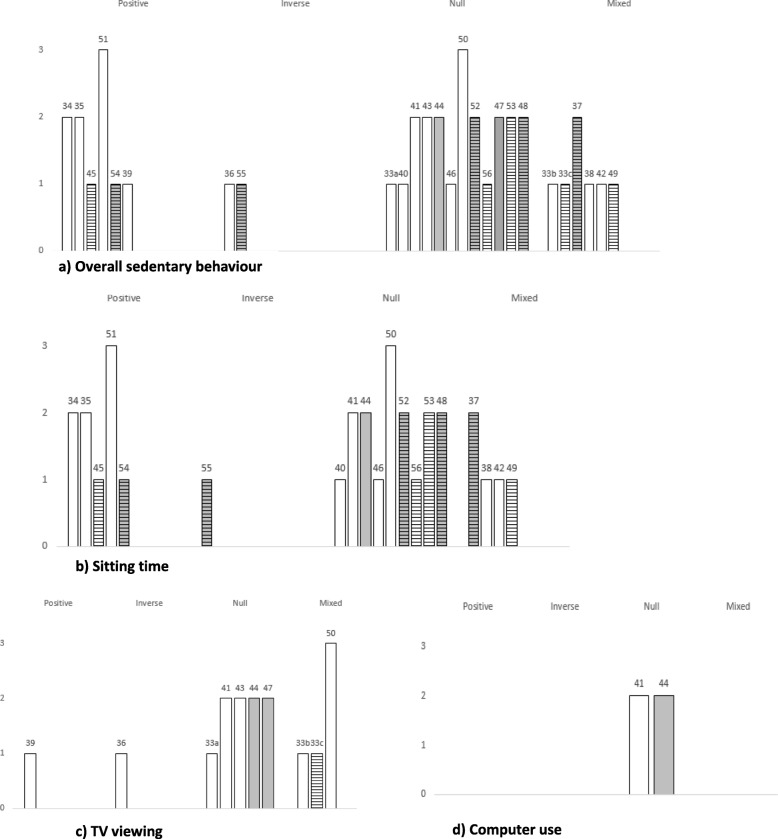
Harvest plot: Evidence for association between indicators of sedentary behaviour and stress. Columns represent individual studies with reference numbers above. Column height represents methodological quality of the study (3 = strong, 2 = moderate, 1 = weak). Shading represents objective measure of stress used (not shaded = subjective measure used). Horizontal lines represent objective measure of sedentary behaviour used (no lines = subjective measure used). Note – for studies that included > 1 model, overall association for those studies was calculated on consistency (≥75%) of findings (see also Table 1)

Figure 2 provides a graphical representation (harvest plots) of the overall evidence. Specifically, of 26 studies reviewed, six (*n* = 1 strong quality [55]; *n* = 2 moderate-quality [34, 35]; *n* = 3 methodologically weak-quality [40, 46, 56]) found a positive association between time spent in any sedentary behaviour and stress (i.e. increased time spent in sedentary behaviour was associated with increased stress). Five of these studies used self-report measures of stress [34, 35, 46, 55, 56], while one study [40] examined salivary cortisol. Eleven studies showed ‘mixed’ findings between sedentary behaviour and stress. Firstly, both positive and null associations were found among nine studies (n = 1 strong- [45]; *n* = 2 moderate- [41, 49]; *n* = 6 weak-quality [33, 38, 44, 51, 57]). Stress was self-reported in eight of these studies [33, 38, 44, 45, 49, 51, 57], while one utilised objective measures (salivary cortisol) [41]).

Both positive and inverse associations between sedentary behaviour and self-reported stress were found in one weak-quality study [37], with the direction of these associations dependent on the source of stress assessed (e.g. total sitting was positively associated with ‘argument-related’ stress, but negatively associated with ‘running late’ stress) [37]. Two weak-quality studies which examined self-reported stress [36] and salivary cortisol [52] respectively, found inverse associations between time spent in sedentary behaviour and stress, whereby increased time in sedentary behaviour was associated with lower levels of stress. Eight remaining studies (*n* = 6 moderate- [39, 43, 48, 50, 53, 54]; *n* = 2 weak-quality [33, 47]) found no association between sedentary behaviour and stress. Stress was self-reported in four of those studies [33, 47, 48, 53]; three used objective measures of stress (hair cortisol [43, 54]; systolic and diastolic blood pressure, heart rate, and salivary cortisol [39]); whilst one study used both a self-report and objective (hair cortisol) measure [50].

#### Best-evidence synthesis

The best-evidence synthesis (adjusting for publication bias and multiple modelling within studies) resulted in the following: Excluding weak studies (since ≥2 moderate/strong quality studies exist), three (25%) (*n* = 1 strong- [55]; *n* = 2 moderate-quality [34, 35]) studies found a positive association between time spent in any sedentary behaviour and stress. One moderate-quality study (9%) [41] showed ‘mixed’ (i.e. positive and null) findings and eight studies (66%) (n = 1 strong- [45]; *n* = 7 moderate- [39, 43, 48–50, 53, 54] found predominately no association between sedentary behaviour and stress. Based on the best-evidence synthesis, there was *insufficient* evidence for an overall relationship between time spent in sedentary behaviour and stress.

### Overall sitting time

Unadjusted results showed that across all studies, there were 60 models that assessed the association between sitting time and stress. This resulted in 23 positive associations (38%); seven inverse associations (12%) and 30 null results (50%).

Specifically, a total of 19 studies (*n* = 2 strong-quality [45, 55]; *n* = 8 moderate-quality [34, 35, 39, 41, 48–50, 54]; *n* = 9 weak-quality [37, 38, 40, 44, 47, 51, 52, 56, 57]) reported on associations between sitting time and stress. Five studies (*n* = 1 strong-quality [55]; *n* = 2 moderate-quality [34, 35]; n = 2 weak-quality [40, 56]) found positive associations between sitting time and stress, i.e. increased sitting time was associated with increased stress. Of those, four employed self-reported measures of stress and non-domain-specific sitting measures, whilst one [40] utilised an objective measure of stress (i.e. salivary cortisol) and a self-reported domain-specific measure of sitting (i.e. occupational sitting) .

One weak-quality study showed an inverse association between sitting time and objectively measured stress (i.e. salivary cortisol) [52]. Four studies (*n* = 1 moderate- [41]; *n* = 3 weak-quality [38, 44, 51]) showed mixed findings. That is, they all included both positive and null results. For example, Lee & Kim (2018) showed a positive association between total and weekday sitting time and stress, but no association between weekend sitting time and stress [44]. Gubelmann et al. (2018) showed a positive association between sitting and diurnal cortisol slope, but no association with mean or awakening cortisol [41]. Nine studies (*n* = 1 strong- [45]; *n* = 5 moderate- [39, 48–50, 54]; *n* = 3 weak-quality [37, 47, 57]) showed predominately no association between sitting time and stress. Of note, however, are the findings from Rebar et al. (2016) [49]. That study compared sitting in three domains (transport-related, leisure-time, occupational) as well as overall sitting. Transport-related sitting time was positively associated with stress, whilst all other models (three of the four [i.e. 75%]) showed no association (and hence the study was labelled as a null overall finding).

#### Best-evidence synthesis

Based on the inconsistent findings across studies (i.e. among the 10 moderate-strong quality studies included, three showed positive associations (30%), one showed mixed (10%), six showed null results (60%)), the best-evidence synthesis resulted in *insufficient evidence* for a relationship between overall sitting time and stress.

### TV viewing

Unadjusted results showed that across all studies, there were 15 models that assessed the association between TV viewing and stress. This resulted in six positive associations (40%); one inverse associations (7%) and eight null associations (53%).

Specifically, ten studies (*n* = 1 strong-quality [45]; *n* = 4 moderate-quality [43, 49, 53, 54]; *n* = 5 weak-quality [33, 36, 46]) investigated associations between TV viewing time and stress. One weak-quality study found a positive association between TV viewing and self-reported stress (i.e. increased TV viewing was associated with greater stress) [46]. Conversely, one weak-quality study found an inverse relationship between TV viewing and self-reported stress (i.e. increased TV viewing was associated with reduced stress) [36]. Mixed results, in this case positive and null associations, were found in two weak- [33] and one strong- [45] quality study. For example, one longitudinal study found no cross-sectional association between TV viewing and perceived stress at baseline, but stress at baseline predicted increased TV viewing at follow-up [45]. Five (*n* = 1 weak-quality [33] and *n* = 4 moderate-quality [43, 49, 53, 54]) studies found no association between TV viewing and stress.

#### Best-evidence synthesis

Among the five moderate-strong quality studies that examined TV viewing, four (80%) showed null associations and therefore based on the best-evidence synthesis there was *strong evidence* for *no association* between TV viewing and stress.

### Computer use

Unadjusted results showed that across all studies, there were two models that assessed the association between computer use and stress, both of which found null associations (100%). Specifically, two moderate-quality studies examined the association between computer use [49, 54] and stress. No association was found between computer use and self-reported stress [49], or computer use and objectively measured stress (hair cortisol) [54] in these studies (100%). Based on the best-evidence synthesis there was *strong evidence* for *no association* between computer use and stress.

### Objectively measured versus self-reported stress

Unadjusted results showed that across all studies, there were 18 models that assessed the association between sedentary behaviour and objectively measured stress. This resulted in two positive associations (11%); six inverse associations (33%) and ten null associations (56%).

Specifically, of the seven studies that used an objective measure of stress (*n* = 2 weak- [40, 52] and *n* = 5 moderate-quality [39, 41, 43, 50, 54]), two reported positive [40] or mixed (in this case positive and null) associations between sedentary behaviour and stress [41] (weak- and moderate quality studies, respectively). Four moderate-quality studies found null associations [39, 43, 50, 54], while one weak-quality study showed inverse associations between sedentary behaviour and stress [52]. Based on the best-evidence synthesis (i.e. four of five moderate-quality studies showed null association (80%), there was *strong evidence for no association* between any type of sedentary behaviour and objectively measured stress.

Unadjusted results showed that across all studies, there were 67 models that assessed the association between sedentary behaviour and self-reported stress. This resulted in 32 positive associations (48%); eight inverse associations (12%) and 27 null results (40%).

Specifically, of the 20 studies that utilised self-report measures of stress, five studies (*n* = 1 strong-quality [55]; *n* = 2 moderate-quality [34, 35]; *n* = 2 weak-quality [46, 56]) showed positive associations, while one weak-quality study showed inverse associations between sedentary behaviour and stress [36]. Five weak-quality studies showed ‘mixed’ (i.e. positive and null) associations between sedentary behaviour and stress [33, 38, 44, 51]). Nine other studies (*n* = 1 strong [45]; *n* = 4 moderate- [48–50, 53]; and *n* = 4 weak-quality [33, 37, 47, 57]) found no associations. Based on the best-evidence synthesis (i.e. three of eight (38%) moderate-strong quality studies showed positive associations, five (62%) showed null associations), there was *insufficient evidence* of a relationship between sedentary behaviour and self-reported stress.

### Objectively measured versus self-reported sedentary behaviour

Unadjusted results showed that across all studies, there were 39 models that assessed the association between objectively measured sedentary behaviour (i.e. device, direct observation) and stress. This resulted in 13 positive associations (33%); seven inverse associations (18%) and 19 null results (49%).

Specifically, ten studies utilised objective measures of sedentary behaviour. Of those, two weak-quality studies showed positive associations [40, 56]. Three studies showed ‘mixed’ (i.e. positive and null) associations between objectively measured sedentary behaviour and stress (*n* = 2 weak- [33, 38] and *n* = 1 moderate-quality [41] studies). One weak-quality study found an inverse association [52]; while four studies (*n* = 3 moderate- [39, 48, 50]; *n* = 1 weak-quality [37] found no associations. Based on the best-evidence synthesis (i.e. of the four moderate-quality studies, one (25%) showed mixed, the other three (75%) showed null associations), there was *strong evidence for no relationship* between objectively measured sedentary behaviour and stress.

Unadjusted results showed that across all studies, there were 39 models that assessed the association between self-reported sedentary behaviour and stress. This resulted in 16 positive associations (41%); one inverse association (3%) and 22 null results (56%).

Specifically, studies that used self-report measures of sedentary behaviour (*n* = 16) yielded the following results. Four studies (*n* = 1 strong- [55]; *n* = 2 moderate- [34, 35]; *n* = 1 weak-quality [46]) found a positive association; four found ‘mixed’ associations (in this case, positive and null; *n* = 1 strong- [45]; *n* = 3 weak-quality [33, 44, 51]; one weak-quality study found an inverse association [36] and seven studies (*n* = 4 moderate-quality [43, 49, 53, 54]; n = 3 weak- [33, 47, 57]) found no association between self-reported sedentary behaviour and stress. Based on the best-evidence synthesis (i.e. of the eight moderate-strong quality studies, three (37%) showed positive, one (13%) showed mixed, four (50%) showed null associations) there was *insufficient evidence* for a positive relationship between self-reported sedentary behaviour and stress.

## Discussion

To the best of our knowledge, this is the first review to systematically synthesise evidence of associations between time spent in sedentary behaviour and stress. Findings showed *insufficient evidence* for the association between overall sedentary behaviour, or sitting time and stress, particularly when using self-report measures of sedentary behaviour or stress. Further, we found *strong evidence* of no association between TV viewing, or computer use and stress; and *strong evidence* of no association between sedentary behaviour and stress when using objective measures of these outcomes.

Given that the findings between overall sitting time and stress were inconsistent, but TV viewing and computer use were strongly not associated with stress, this may indicate that the type of sedentary behaviour could potentially play a role in the link with stress. It is possible that time spent watching TV and using computers may have confounded the association between sitting time (which includes, but is not limited to, both of these behaviours) and stress. It may be that other sedentary behaviour indicators (e.g. smartphone and tablet use) that were not captured in these studies, yet contribute to overall sitting time, may be subsequently linked to greater stress. However, no studies utilised measures of these more modern electronic devices (i.e. smartphones/tablets) and therefore this is a key area identified as requiring further research. It has been hypothesised that engaging in high levels of social media use (predominately undertaken whilst using modern electronic devices such as smartphones or tablets) may adversely impact mental health outcomes (e.g. anxiety, stress, depressive symptoms, including suicidal behaviours [23]), particularly since social media use has been associated with feelings of “addiction” and loss of sleep [58]. Further, it may be that watching TV or using computers (which are likely to be used for both leisure and occupational purposes) are not detrimental to mental health, given that some studies have suggested TV viewing may be used by adults experiencing depressive symptoms as a time to relax and ‘switch off’ [59].

Another finding of this review was that although there was *insufficient evidence* for the association between sedentary behaviour and stress when using self-report measures, there was strong evidence for *no association* between sedentary behaviour and stress when using objective measures of these outcomes. There is mixed evidence regarding the relationship between self-report and objectively-assessed stress. Whilst some studies have shown that self-report retrospective measures of stress are associated with objective measures (e.g. heart rate variability) of stress [60], other studies have shown that self-report measures of stress, assessed only contemporaneously but not retrospectively, are associated with objective measures (e.g. salivary cortisol) [61]. Although self-report measures can be subject to bias and recall error, objective measures also present limitations. For example, salivary cortisol levels fluctuate diurnally and can be influenced by other factors such as age or sleep [62]. Similarly, current objective, specifically device-assessed, measures of sedentary behaviour (e.g. inclinometers) are limited by the lack of distinction between the *type* (e.g. computer use, electronic device use, TV viewing) and *context* (e.g. work-related emails, social media use, online shopping) of activity being performed. Given that the *type* and/or *context* of sedentary behaviour could potentially be a key factor in the relationship with stress, rather than the sitting posture itself (as indicated by previous research examining associations between sedentary behaviour and other mental health outcomes [21, 22]), further studies utilising objective behaviour-specific measures of sedentary behaviour, which capture type and context, are required. This could include utilising existing technology such as smartphone apps (e.g. *Moment app*) that track the duration of time spent using electronic devices, which currently has not been done in studies examining the link between sedentary behaviour and mental health.

Only one study [49] examined associations between different domains of sitting time (i.e. transport, leisure, occupational) and stress, and results indicated some domains of sitting (in this case transport-related sitting) were more strongly associated with stress than other domains (i.e. leisure, occupational). These findings contrast those of another study that investigated the link between occupational sitting time and stress [40], which found a positive association. Although the domain of sedentary behaviour could potentially play a role in the relationship with stress, currently little research distinguishing *domains* of sedentary behaviour exists. This is another consideration when selecting measures for future studies, given that sitting at the computer for work purposes could potentially elicit differing stress responses compared to sitting at the computer for leisure purposes. To further add to the complexity of this research area, it has been suggested that standing (as opposed to sitting) whilst engaging in such screen-based behaviours (using a sit-stand workstation for example) may reduce the risk of poor mental health [63]. However, as previously described, the *type and context* of the sedentary behaviour is likely to be a key determinant in the relationship with stress, and is likely a more important determinant than the actual sitting/standing posture itself [49].

Studies included in this review were limited by other factors including: more than half (54%) were rated as weak-quality and 62% utilised cross-sectional study designs in which temporal associations or cause and effect were unable to be determined. Studies used varied measures of sedentary behaviour and stress, which limits the ability to directly compare findings, and it was not possible to conduct a meta-analysis [29, 30]. A number of studies did not report on the reliability or validity of measures of sedentary behaviour and/or stress used. Further, there is a lack of evidence to suggest whether prolonged, uninterrupted sitting has a different effect or association on stress, compared to if one regularly breaks up their sitting time. In addition, no studies examined interactions between specific sedentary behaviours (e.g. TV viewing, computer use, electronic device use) and total sedentary time. Thus, it could be possible that the effects of, for example, TV viewing would be dependent on the total amount of sedentary behaviour (e.g., with a low total amount of sedentary behaviour the effects of TV viewing could be beneficial but with high total amount of sedentary behaviour the effects of TV viewing could be harmful). This is worthy of further examination.

## Conclusions

Although limited (i.e. predominately weak-quality, cross-sectional) research has explored associations between sedentary behaviour and stress, this review consolidates the existing evidence and found insufficient (due to conflicting results) evidence for the association between sedentary behaviour and overall sitting time and stress. Further there was strong evidence for no association between other specific types of sedentary behaviour (e.g. TV viewing, computer use) and stress. This review and the results presented provides preliminary information to question why we should investigate the link between sedentary behaviour and stress and highlights the importance of the activity that is being undertaken whilst the sitting is occurring (rather than the sitting itself). High-quality longitudinal/interventional research is required to confirm findings and determine the direction of associations between different types, contexts and domains of sedentary behaviour and stress.

## Supplementary information

**Additional file 1: Table S1.** Sedentary behaviour and stress systematic review databases and search terms

**Additional file 2: Table S2.** Methodological quality assessment of studies included in the present review

## Data Availability

The datasets supporting the conclusions of this article are included within the article (and Additional files).

## Abbreviations

CBS: Chronic Burden Scale
DASS: Depression, Anxiety and Stress Scale
DSI: Daily Stress Inventory
ERIS: Effort Reward Imbalance Scale
LEI: Life Events Inventory
PRISMA: Preferred reporting items for systematic reviews
PSQYW: Perceived Stress Questionnaire for Young Women
PSS: Cohen Perceived Stress Scale
SB: Sedentary behaviour
TSS: Traumatic Stress Schedule
TV: Television viewing
UK: United Kingdom
US: United States
