# Supplementary material for: The association between sedentary behaviour and indicators of stress: a systematic review
Source: BMC Public Health. 2019 Oct 23;19:1357. doi: 10.1186/s12889-019-7717-x (PMC6813058; doi:10.1186/s12889-019-7717-x)
Supplement: Supplementary file 1 — Additional file 1: Table S1. Sedentary behaviour and stress systematic review databases and search terms [file 12889_2019_7717_MOESM1_ESM.docx]

**Additional file 1: Table S1:** Sedentary behaviour and stress systematic review databases and search terms

| **Database** | **Search terms** |
| --- | --- |
| **MEDLINE/MEDLINE COMPLETE** | 1. TI “Sedentary behav*” OR AB “Sedentary behav*” OR TI “screen time” OR AB “screen time” OR TI “screen based” OR AB “screen based” OR TI “TV” OR AB “TV” OR TI “television” OR AB “television” OR TI “computer” OR AB “computer” OR TI “Electronic device” OR AB “Electronic device” OR TI “video game” OR AB “video game” OR TI “smartphone” OR AB “smartphone” OR TI “smart phone” OR AB “smart phone” OR TI “sitting” OR AB “sitting” OR TI “passive transport” OR AB “passive transport” OR TI “tablet” OR AB “tablet” |
|  | 1. TI “Stress*” OR AB “Stress*” OR MH “stress, psychological” OR TI “cortisol” OR AB “cortisol” OR TI “adrenocortical” OR AB “adrenocortical” OR TI “glucocortico*” OR AB “glucocortico*” OR TI “ACTH” OR AB “ACTH” OR TI “adrenocorticotropin hormone” OR AB “adrenocorticotropin hormone” OR TI “adrenocorticotropic hormone” OR AB “adrenocorticotropic hormone” OR TI “corticotropin” OR AB “corticotropin” OR TI “adrenocorticotrophin hormone” OR AB “adrenocorticotrophin hormone” OR TI “adrenocorticotrophic hormone” OR AB “adrenocorticotrophic hormone” OR TI “corticotrophin” OR AB “corticotrophin” |
|  | 1. 1 AND 2 |
|  | 1. Limited to January 1^st^ 1990 to September 9^th^ 2019, English language only |
|  |  |
| **CINAHL** | 1. TI “Sedentary behav*” OR AB “Sedentary behav*” OR TI “screen time” OR AB “screen time” OR TI “screen based” OR AB “screen based” OR TI “TV” OR AB “TV” OR TI “television” OR AB “television” OR TI “computer” OR AB “computer” OR TI “Electronic device” OR AB “Electronic device” OR TI “video game” OR AB “video game” OR TI “smartphone” OR AB “smartphone” OR TI “smart phone” OR AB “smart phone” OR TI “sitting” OR AB “sitting” OR TI “passive transport” OR AB “passive transport” OR TI “tablet” OR AB “tablet” |
|  | 1. TI “Stress*” OR AB “Stress*” OR MH “stress, psychological” OR TI “cortisol” OR AB “cortisol” OR TI “adrenocortical” OR AB “adrenocortical” OR TI “glucocortico*” OR AB “glucocortico*” OR TI “ACTH” OR AB “ACTH” OR TI “adrenocorticotropin hormone” OR AB “adrenocorticotropin hormone” OR TI “adrenocorticotropic hormone” OR AB “adrenocorticotropic hormone” OR TI “corticotropin” OR AB “corticotropin” OR TI “adrenocorticotrophin hormone” OR AB “adrenocorticotrophin hormone” OR TI “adrenocorticotrophic hormone” OR AB “adrenocorticotrophic hormone” OR TI “corticotrophin” OR AB “corticotrophin” |
|  | 1. 1 AND 2 |
|  | 1. Limited to January 1^st^ 1990 to September 9^th^ 2019, English language only |
|  |  |
| **PsychINFO** | 1. TI “Sedentary behav*” OR AB “Sedentary behav*” OR TI “screen time” OR AB “screen time” OR TI “screen based” OR AB “screen based” OR TI “TV” OR AB “TV” OR TI “television” OR AB “television” OR TI “computer” OR AB “computer” OR TI “Electronic device” OR AB “Electronic device” OR TI “video game” OR AB “video game” OR TI “smartphone” OR AB “smartphone” OR TI “smart phone” OR AB “smart phone” OR TI “sitting” OR AB “sitting” OR TI “passive transport” OR AB “passive transport” OR TI “tablet” OR AB “tablet” |
|  | 1. TI “Stress*” OR AB “Stress*” OR MA “stress, psychological” OR TI “cortisol” OR AB “cortisol” OR TI “adrenocortical” OR AB “adrenocortical” OR TI “glucocortico*” OR AB “glucocortico*” OR TI “ACTH” OR AB “ACTH” OR TI “adrenocorticotropin hormone” OR AB “adrenocorticotropin hormone” OR TI “adrenocorticotropic hormone” OR AB “adrenocorticotropic hormone” OR TI “corticotropin” OR AB “corticotropin” OR TI “adrenocorticotrophin hormone” OR AB “adrenocorticotrophin hormone” OR TI “adrenocorticotrophic hormone” OR AB “adrenocorticotrophic hormone” OR TI “corticotrophin” OR AB “corticotrophin” |
|  | 1. 1 AND 2 |
|  | 1. Limited to January 1^st^ 1990 to September 9^th^ 2019, English language only |
|  |  |
| **SPORTDiscus** | 1. TI “Sedentary behav*” OR AB “Sedentary behav*” OR TI “screen time” OR AB “screen time” OR TI “screen based” OR AB “screen based” OR TI “TV” OR AB “TV” OR TI “television” OR AB “television” OR TI “computer” OR AB “computer” OR TI “Electronic device” OR AB “Electronic device” OR TI “video game” OR AB “video game” OR TI “smartphone” OR AB “smartphone” OR TI “smart phone” OR AB “smart phone” OR TI “sitting” OR AB “sitting” OR TI “passive transport” OR AB “passive transport” OR TI “tablet” OR AB “tablet” |
|  | 1. TI “Stress*” OR AB “Stress*” OR TI “stress, psychological” OR AB “stress, psychological” OR TI “cortisol” OR AB “cortisol” OR TI “adrenocortical” OR AB “adrenocortical” OR TI “glucocortico*” OR AB “glucocortico*” OR TI “ACTH” OR AB “ACTH” OR TI “adrenocorticotropin hormone” OR AB “adrenocorticotropin hormone” OR TI “adrenocorticotropic hormone” OR AB “adrenocorticotropic hormone” OR TI “corticotropin” OR AB “corticotropin” OR TI “adrenocorticotrophin hormone” OR AB “adrenocorticotrophin hormone” OR TI “adrenocorticotrophic hormone” OR AB “adrenocorticotrophic hormone” OR TI “corticotrophin” OR AB “corticotrophin” |
|  | 1. 1 AND 2 |
|  | 1. Limited to January 1^st^ 1990 to September 9^th^ 2019, English language only |
|  |  |
| **EMBASE** | 1. ‘Sedentary behav*’:ab,ti OR ‘screen time’:ab,ti OR ‘screen based’:ab,ti OR ‘TV’:ab,ti OR ‘television’:ab,ti OR ‘computer’:ab,ti OR ‘Electronic device’:ab,ti OR ‘video game’:ab,ti OR ‘smartphone’:ab,ti OR ‘smart phone’:ab,ti OR ‘sitting’:ab,ti OR ‘passive transport’:ab,ti OR ‘tablet’:ab,ti |
|  | 1. ‘Stress*’:ab,ti OR ‘stress, psychological’:ab,ti OR ‘cortisol’:ab,ti OR ‘adrenocortical’:ab,ti OR ‘glucocortico*’:ab,ti OR ‘ACTH’:ab,ti OR ‘adrenocorticotropin hormone’:ab,ti OR ‘adrenocorticotropic hormone’:ab,ti OR ‘corticotropin’:ab,ti OR ‘adrenocorticotrophin hormone’:ab,ti OR ‘adrenocorticotrophic hormone’:ab,ti OR ‘corticotrophin’:ab,ti |
|  | 1. 1 AND 2 |
|  | 1. Limited to January 1^st^ 1990 to September 9^th^ 2019, English language only |
